# Forced eruption in impacted teeth: analysis of failed cases and outcome of re-operation

**DOI:** 10.1186/s12903-024-03963-x

**Published:** 2024-02-20

**Authors:** Jaeyeon Kim, Seoyeon Jung, Kee-Joon Lee, Hyung-Seog Yu, Wonse Park

**Affiliations:** 1 Department of Advanced General Dentistry, College of Dentistry, Yonsei University, Seodaemun-gu, Seoul, South Korea; 2 Institute for Innovation in Digital Healthcare, Yonsei University, Seodaemun-gu, Seoul, South Korea; 3 Department of Dental Education, College of Dentistry, Yonsei University, Seodaemun‑gu, Seoul, South Korea; 4 Department of Orthodontics, Institute of Craniofacial Deformity, College of Dentistry, Yonsei University, Seodaemun‑gu, Seoul, South Korea

**Keywords:** Orthodontic treatment, Re-operation, Apex formation, Tooth positioning, Rotation

## Abstract

**Background:**

Forced eruption of an impacted tooth usually requires surgical and orthodontic interventions to successfully bring the tooth into the dental arch. The clinical time required for a forced eruption is difficult to predict before treatment begins and success rates are affected by several factors before and after an eruption. This study was conducted to identify factors that affect the success of forced eruption, the duration of orthodontic treatment of impacted teeth, and the reasons for re-operation and forced eruption failure in a various teeth and cases.

**Methods:**

In this retrospective study, the records regarding the forced eruption of 468 teeth in 371 patients from June 2006 to May 2020 at the Advanced General Dentistry Department of Yonsei University Dental Hospital were initially examined. The records of 214 teeth in 178 patients who completed orthodontic treatment were included in the analysis. Data on patient demographics, tooth characteristics, orthodontic treatment duration, re-operations, and failures were collected from electronic medical records.

**Results:**

There was a significant difference in age between the success and failure forced eruption. Factors significantly affecting treatment duration were apex formation, position, rotation, and re-operation. Re-operation had a 96% success rate. The average orthodontic treatment duration was 29.99 ± 16.93 months, but the average orthodontic treatment duration for teeth that undergone re-operation was 20.36 ± 11.05 months, which was approximately 9 months shorter. Additionally, there was an interaction effect between rotation and re-operation on the duration of orthodontic treatment. The causes for failure of forced eruption in 6 cases were ankyloses (3 cases), incomplete alignment with the normal dental arch (2 cases), and a significant deviation in the impacted tooth’s location (1 case).

**Conclusions:**

To increase the success rate of forced eruption, age should be considered as a priority, and in order to predict the treatment period, the apex formation status, position in the arch, and rotation should be considered in addition to age. When determining re-operation, considering factors such as ankylosis, root curvature, and apex formation can help in the success of orthodontic treatment.

**Supplementary Information:**

The online version contains supplementary material available at 10.1186/s12903-024-03963-x.

## Introduction

The most frequently impacted teeth are third molars (91.6%), followed by canines (5.3%) and premolars (1.6%) [1]. Impacted teeth can be caused by a variety of factors, including mechanical obstruction, malpositioning of the tooth bud, dental cysts, and genetic factors, such as craniofacial dysostosis, osteopetrosis, hypothyroidism and ankylosis [2–5]. Failure of early diagnosis and treatment of impacted teeth can lead to serious damage, such as external resorption of adjacent teeth, esthetic problems, reduced dental arches, and increased follicular cyst formation, which can lead to tooth loss and periodontal involvement [6–9].

Impacted teeth require complex therapeutic management that is successful if forced eruption and subsequent alignment lead the tooth to the correct position in the dental arch [10]. Forced eruption of an impacted tooth usually requires surgical and orthodontic interventions to successfully bring the tooth into the dental arch [11]. Forced eruption of impacted teeth takes longer than most other orthodontic treatments and often involves the entire orthodontic treatment duration rather than just the duration until the impacted tooth is aligned with the dental arch [12, 13]. The clinical time required for a forced eruption is difficult to predict before treatment begins [14].

The success rates of forced eruption are affected by several factors before and after an eruption. Before eruption, the success rate is affected by the age, position and orientation of the impacted tooth [15]. After forced eruption, the success rate is affected by impacted tooth displacement, root curvature, ankyloses, changes in gingival tissue, oral health management ability, patient cooperation, reduction in secondary effects due to the fixation force of the orthodontic appliance, and the effect of removable devices [16, 17]. Therefore, treatment method selection requires careful consideration of these factors.

Several studies have been conducted on the success rates and orthodontic treatment duration of forced eruption. However, most of these studies are limited to the maxillary canines [18–20]. Therefore, this study aimed to investigate the factors affecting the success of forced eruption, duration of orthodontic treatment of impacted teeth, and causes of re-operation and forced eruption failure in a various teeth and cases.

## Materials and methods

We screened electronic medical records, including dental radiographs, of forced eruption procedures for 468 teeth in 371 patients conducted in the Department of Advanced General Dentistry at Yonsei University Dental Hospital and orthodontic treatment completed at the Department of Orthodontics at Yonsei University Dentistry Hospital between June 2006 and May 2020. The study protocol was approved by the Institutional Review Board of the Yonsei University Dental Hospital (approval number:2-2020-0073). Patient data were anonymized, and the requirement for obtaining written informed consent was waived because of the retrospective nature of this study. The study was conducted in accordance with the principles of the Declaration of Helsinki.

### Inclusion/Exclusion Criteria

The inclusion criteria were as follows: (1) patients who underwent forced eruption with at least one impacted tooth and received an orthodontic device at the Department of Advanced General Dentistry at Yonsei University Dental Hospital; (2) patients who completed orthodontic treatment; (3) patients with complete treatment history (surgical exposure and button attachment date, banding and bonding date, full arch or sectional fixation date, debanding and debonding date). The exclusion criteria were as follows: (1) patients who underwent forced eruption of third molars; (2) patients with tooth eruption disorders such as tumors, odontoma or cysts.

### Data collection

Data were collected regarding patients’ age, sex, whether the tooth that had undergone forced eruption was maxillary or mandibular teeth, total orthodontic treatment duration, and whether the procedure had failed. The apex formation, impacted tooth position and rotation at the time of button attachment were analyzed using a panoramic view and cone-beam CT. For participants who underwent re-operation after the first forced eruption treatment, the cause for re-operation was also investigated. In addition, in cases of forced eruption treatment failure, apex formation, tooth position and impaction, whether re-operation was required, cause of failure, and treatment outcomes were investigated.

Age was defined as the difference between the year in which the patients received forced eruption treatment and their year of birth. The total orthodontic treatment duration was defined as the difference between the month in which the orthodontic device was removed and the retainer was fixed and the month in which surgical excision and button attachment occurred.

Forced eruption was defined as successful when orthodontic traction and a stable occlusal relationship were obtained, and as having failed when the tooth was partially moved after forced eruption but not completely aligned with the dental arch, tooth eruption was incomplete, ankylosis occurred, or the tooth was extracted because forced eruption traction was not possible.

### Data analysis

The data were analyzed by tooth because the likelihood of success and the treatment duration were determined by differences in tooth position and traction path. Descriptive data are expressed as n (%) or mean ± standard deviation. A normality test was performed to compare categorical variables between the forced eruption success group and the failure group, and considering that the data did not meet the normality assumption, fisher’s exact test and Mann-Whitney U test, a non-parametric statistic, was used. Univariate and multivariate binary logistic regression analyses were used to evaluate the relationship between demographic/clinical characteristics and the duration of orthodontic treatment for impacted teeth, and two-way ANOVA was used to evaluate the correlation between various factors and re-operation according to the duration of orthodontic treatment. Statistical significance was set at *p* < 0.05 for all analyses. All statistical tests were performed using SPSS statistical software (SPSS for Windows, version 25; SPSS Inc., Chicago, IL, U.S.A).

## Results

According to the inclusion and exclusion criteria, 214 teeth in 178 patients were selected from 468 teeth in 371 patients. The demographic and clinical characteristics of the 214 teeth are presented in Table 1. Of these, 125 (58.4%) were female with a mean age of 15.36 ± 6.20 years. Maxillary teeth (63.6%) and single-rooted teeth (75.2%) were the most commonly impacted. The apex was closed in 55.1% of cases, 57.5% were positioned in the arch, and 58.4% mesial/distal rotation. Re-operation was not performed in 88.3% of cases, and successful forced eruption was 97.2%. The average orthodontic treatment duration was 29.99 ± 16.93 months.

**Table 1:** Demographics and clinical characteristics

Characteristics		Overall (*n* = 214)
Sex		
	Female	125 (58.4)
	Male	89 (41.6)
Age		15.36 ± 6.20
Location		
	Maxillary	136 (63.6)
	Mandibular	78 (36.4)
Root		
	Single	161 (75.2)
	Double	44 (20.6)
	Multiple	9 (4.2)
Apex formation		
	Open	96 (44.9)
	Closed	118 (55.1)
Position		
	Line in arch	123 (57.5)
	Palatal/Buccal	91 (42.5)
Rotation		
	Vertical	44 (20.6)
	Mesial/Distal	125 (58.4)
	Horizontal	45 (21.0)
Re-operation		
	Not performed	189 (88.3)
	Performed	25 (11.7)
Success or Failure		
	Success	208 (97.2)
	Failure	6 (2.8)
Duration		29.99 ± 16.93
	Within 2 years	94 (43.9)
	More than 2 years	120 (56.1)

Values are *n* (%), mean ± standard deviation, as indicated.

### Factors affecting success or failure of forced eruption and duration of orthodontic treatment

There was significant difference in age between the success and failure groups of forced eruption. In the success group, those aged 10–19 years accounted for the most at 94.0%. and in the failure group, those aged 10 to 19 and 20 to 29 accounted for 33.3%, respectively, and those aged 30 or older accounted for 16.7% (Table 2).

**Table 2:** Factors that were correlated with forced eruption success or failure

Variables		Overall	Success (*n* = 208)	Failure (*n* = 6)	*P*
Sex					0.404
	Female	125 (58.4)	120 (57.7)	5 (83.3)	
	Male	89 (41.6)	88 (42.3)	1 (16.7)	
Age					0.030*
	0–9	18 (8.4)	17 (8.2)	1 (16.7)	
	10–19	156 (72.9)	154 (94.0)	2 (33.3)	
	20–29	36 (16.8)	34 (16.3)	2 (33.3)	
	30 and above	4 (1.9)	3 (1.4)	1 (16.7)	
Location					1.000
	Maxillary	136 (63.6)	132 (63.5)	4 (66.7)	
	Mandibular	78 (36.4)	76 (36.5)	2 (33.3)	
Root					1.000
	Single	161 (75.2)	156 (75.0)	5 (83.3)	
	Double	44 (20.6)	43 (20.7)	1 (16.7)	
	Multiple	9 (4.2)	9 (4.3)	.	
Apex formation					0.693
	Open	96 (44.9)	94 (45.2)	2 (33.3)	
	Closed	118 (55.1)	114 (54.8)	4 (66.7)	
Position					0.405
	Line in arch	123 (57.5)	121 (58.2)	2 (33.3)	
	Palatal/Buccal	91 (42.5)	87 (41.8)	4 (66.7)	
Rotation					0.064
	Vertical	44 (20.6)	42 (20.2)	2 (33.3)	
	Mesial/Distal	125 (58.4)	124 (59.6)	1 (16.7)	
	Horizontal	45 (21.0)	42 (20.2)	3 (50.0)	
Re-operation					0.53
	Not performed	189 (88.3)	184 (88.5)	5 (83.3)	
	Performed	25 (11.7)	24 (11.5)	1 (16.7)	

Values are *n* (%), mean ± standard deviation, as indicated

Mann-Whitney U test was used for continuous variables such as age, and Fisher’s exact test was used for categorical variables such as sex, location, root, apex formation, position, rotation and re-operation

In the univariate logistic regression analysis presented in Table 3, apex formation is open compared to closed (OR, 2.423; 95% CI, 1.161–5.054), position is line in arch compared to palatal/buccal (OR, 5.060; 95% CI, 2.190-11.693), rotation is horizontal compared to vertical (OR, 2.965; 95% CI, 1.019–8.627), and re-operation is performed rather than not performed (OR,0.137; 95% CI, 0.046–0.409) was a significant association with the group that completed orthodontic treatment within 2 years. Among the factors evaluated in univariate analysis, apex formation, position, rotation, and re-operation were included in multivariate logistic regression analysis. In the multivariate logistic regression model, apex formation (OR, 1.967; 95% CI, 1.062–3.643), tooth position in the arch (OR, 3.903; 95% CI, 2.003–7.606), horizontal rotation (OR, 3.628; 95% CI, 1.297–10.147), and the re-operation (OR, 0.154; 95% CI, 0.054–0.437) were predictors of orthodontic treatment duration for more than 2 years.

### Causes and treatment duration of re-operation

Twenty-five teeth underwent re-operation (Table 4). There were 13 (52.0%) of maxillary canines and 6 cases (24.0%) of mandibular molars. The average duration from the first button attachment to the re-operation was 6.20 ± 3.82 months. The orthodontic treatment duration was 20.36 ± 11.05 months. The most common cause for re-operation was button, ligature, or wire detachment, which was observed in 13 cases (52.0%). The apex formation was open in 7 cases (28.0%), closed in 18 cases (72.0%), root curvature was straight in 19 cases (76.0%), curved of orthodontic movement in 3 cases (12.0%), and Curved against orthodontic movement was 3 cases (12.0%). The success rate for teeth that underwent re-operation was 96%, and the mandibular canine tooth failed to underwent re-operation.

**Table 3:** Univariate and multivariate logistic regression models of duration of orthodontic treatment

Variables	Within 2 years (n = 94)	More than 2 years (n = 120)	Univariate logistic regression analysis		Multivariate logistic regression analysis	
			OR (95% CI)	*P*	OR (95% CI)	*P*
Sex						
Female	55 (58.5)	70 (58.3)	1			
Male	39 (41.5)	50 (41.7)	1.520 (0.787–2.935)	0.212		
Age						
0–9	7 (7.4)	11 (9.2)	1			
10–19	70 (74.5)	85 (71.4)	1.255 (0.380–3.947)	0.734		
20–29	14 (14.9)	22 (18.5)	0.980 (0.239–4.012)	0.977		
30 and above	3 (3.2)	1 (0.8)	0.776 (0.052–11.576)	0.854		
Location						
Maxillary	51 (54.3)	85 (70.8)	1			
Mandibular	43 (45.7)	35 (29.2)	0.635 (0.281–1.432)	0.274		
Root						
Single	67 (71.3)	94 (78.3)	1			
Double	22 (23.4)	22 (18.3)	1.930 (0.749–4.969)	0.173		
Multiple	5 (5.3)	4 (3.3)	0.997 (0.223–4.460)	0.997		
Apex formation						
Open	51 (54.3)	45 (37.5)	1		1	
Closed	43 (45.7)	75 (62.5)	2.423 (1.161–5.054)	0.018*	1.967 (1.062–3.643)	0.032*
Position						
Line in arch	71 (75.5)	52 (43.3)	1		1	
Palatal/Buccal	23 (24.5)	68 (56.7)	5.060 (2.190–11.693)	0.000***	3.903 (2.003–7.606)	0.000***
Rotation						
Vertical	27 (28.7)	17 (14.2)	1		1	
Mesial/Distal	57 (60.6)	68 (56.7)	1.433 (0.643–3.191)	0.379	1.765 (0.816–3.816)	0.149
Horizontal	10 (10.6)	35 (29.2)	2.965 (1.019–8.627)	0.046*	3.628 (1.297–10.147)	0.014*
Re-operation						
Not performed	76 (80.9)	113 (94.2)	1		1	
Performed	18 (19.1)	7 (5.8)	0.137 (0.046–0.409)	0.000***	0.154 (0.054–0.437)	0.000***

Abbreviations: OR, Odds Ratio; CI, Confidence Interval

*p < 0.05; **p < 0.01, ***p = 0.000

**Table 4:** Patients with re-operation data (*n* = 25)

Teeth		Duration (month)		Cause				Apex formation		Root curvature			Success	Total
		1st to 2nd^a^	1st to debond^b^	Button, ligature or wire detachment	Button repositioning	Re-opening with ostectomy	Adjunctive soft tissue	Open	Closed	Straight	Curved of orthodontic movement	Curved against orthodontic movement		
Maxillary														
	Incisor	7	40	1	.	.	.	.	1	1	.	.	1	1
	Canine	6.2	28.8	4	4	3	2	4	9	10	1	2	13	13
Mandibular														
	Canine	4	17.5	3^c^	.	.	.	1	2^c^	2	.	1^c^	2	3
	Premolar	8	69	1	.	1	.	1	1	1	1	.	2	2
	Molar	6	20.8	4	1	.	1	1	5	5	1	.	6	6
Total (%)		6.20	20.36	13 (52.0)	5 (20.0)	4 (16.0)	3 (12.0)	7 (28.0)	18 (72.0)	19 (76.0)	3 (12.0)	3 (12.0)	24 (96.0)	**25 (100)**

^a^ The duration from button attachment, the first stage of forced eruption, to re-operation

^b^ The duration from the first button attachment to the entire course of orthodontic treatment including re-operation

^c^ Case of failure after re-operation

**Fig. 1:**
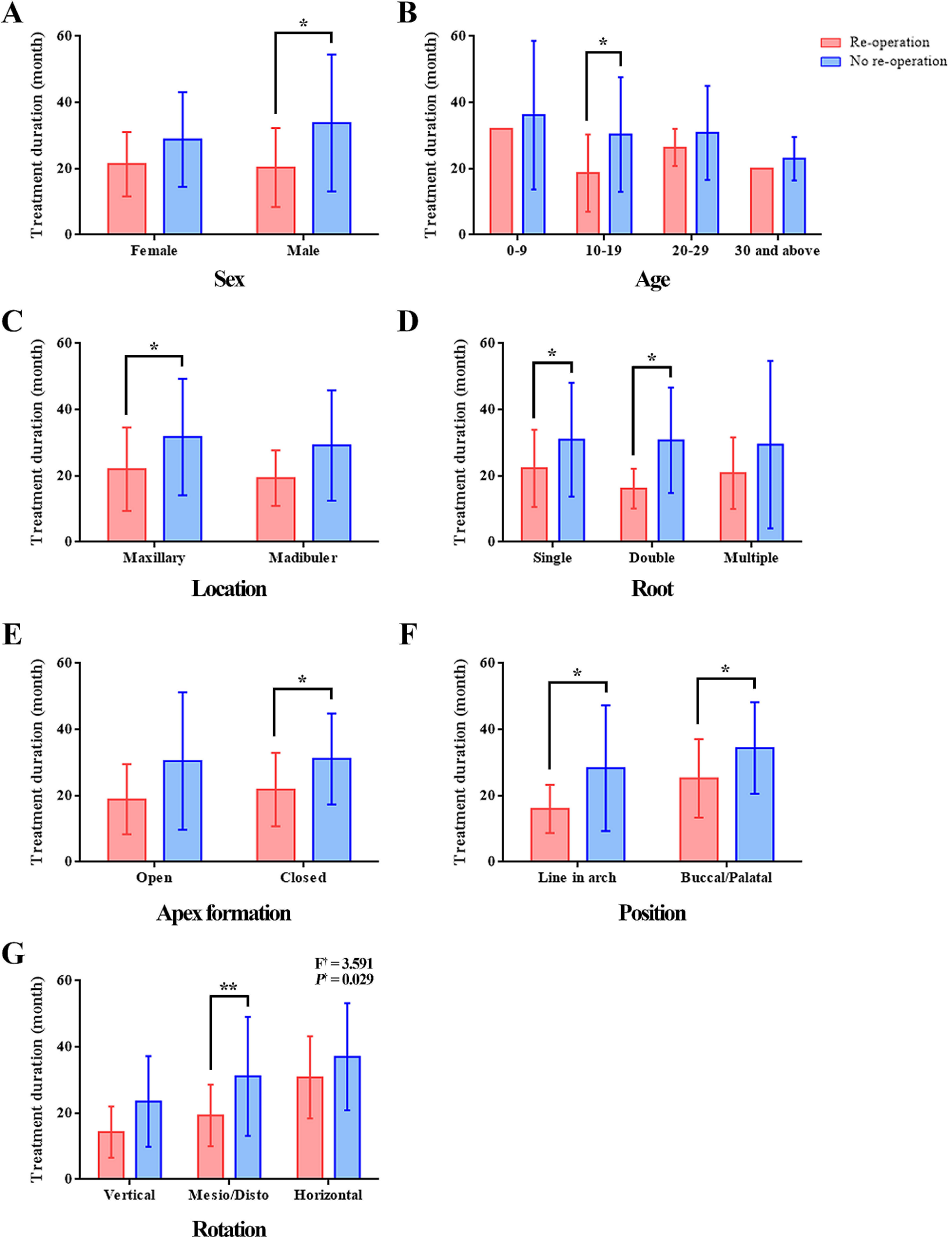
Comparison of interactions between factors affecting the re-operation on the duration of orthodontic treatment. **(A)** Sex, **(B)** Age, **(C)** Location, **(D)**, Root, **(E)** Apex formation, **(F)** Position, **(G)** Rotation. ^†^ Changes in orthodontic treatment duration according to re-operation and rotation; *P < 0.05, **P < 0.01

In the group that undergone re-operation, the orthodontic treatment duration for vertical, mesio/disto, and horizontal rotation was 14.25 ± 7.72 months, 19.31 ± 9.29 months, and 30.80 ± 12.44 months, respectively, and in the group without re-operation, it was 23.54 ± 13.67 months, 31.11 ± 17.98 months, and 37.05 ± 16.17 months, respectively (Fig. 1). There was an interaction effect between rotation and re-operation on the duration of orthodontic treatment.

In addition, through comparison between individual factors, the variables male, 10–19 years old, maxilla, single or double root, teeth with closed apex formation, line in arch or buccal/palatal position, and mesio/disto rotation were found to have a significantly shorter orthodontic treatment duration in the group that undergone re-operation compared to the group that did not re-operation. The case of a patient who undergone re-operation is described in supplemental file 1.

### Case of forced eruption failure

In the examined cases of forced eruption, the age ranged from 9 to 45 years old, and women accounted for the most cases, with 5 cases. The most common cases were maxillary (4 cases), canine (4 cases), and apex closed (5 cases) (Table 5). The primary causes of failure included ankylosis in three cases, incomplete alignment with the normal dental arch in two cases, and a significant deviation in the impacted tooth’s location in one case. A detailed description of the case is provided in Supplemental files 1.

**Table 5:** Failure cases of patients with forced eruption

Case No.	Age (years)	Sex	Location/Teeth	Apex formation	Position	Impacted Direction	Re-operation	Cause of failure	Outcome	Duration (Month)	
										Forced eruption	Total Treatment
1	24	Female	Maxillary/Canine	Closed	Buccal	Horizontal	No	Ankylosis	Extraction	3	32
2	12	Female	Maxillary/Premolar	Closed	Line in arch	Vertical	No	Ankylosis	Extraction	9	54
3	9	Female	Mandibular/Molar	Open	Line in arch	Vertical	No	Ankylosis	Extraction	12	41
4	29	Female	Mandibular/Canine	Closed	Palatal	Horizontal	Yes	Incomplete alignment	Extraction & implant	13	33
5	45	Male	Maxillary/Canine	Closed	Palatal	Horizontal	No	Incomplete alignment	Extraction & implant	18	24
6	13	Female	Maxillary/Canine	Closed	Buccal	Vertical	No	Too far from the dental arch	Extraction		19

^a^ The duration from button attachment, the first stage of forced eruption, to re-operation

^b^ The duration from the first button attachment to the entire course of orthodontic treatment including re-operation

^c^ Case of failure after re-operation

## Discussion

This retrospective study was conducted using the records of patients who underwent forced eruption at the Department of Advanced General Dentistry at Yonsei University Dental Hospital over 15 years. Various studies have investigated the duration and risk factors of forced eruption treatment of impacted canines, which occurs in 0.92–2.4% of canines [21]. Most studies are limited in that they focus on forced eruption specific to canine and there is a lack of studies of forced eruption of other teeth [22]. Therefore, in this study, all 214 impacted teeth that had undergone forced eruption except the third molar were investigated.

Becker et al. reported that the success rate for patients over 30 years of age was 41%, whereas that for patients aged 20–30 years was 100%, and that there was a significant relationship between treatment difficulty and age [23]. Consistent with these findings, Potrubacz et al. reported that the shortest treatment duration was observed in patients aged 11–12 years [24]. In this study, the maximum age at which the forced eruption was successful was 51 years. The age of the failure group increased more than five years compared to the success group. In this study, age also showed a significant difference in success and failure. However, there was no significant difference depending on the period. This may be because the sample size in this study was small, and while most related studies focused only on impacted canines, this study targeted all teeth.

This study showed that apex formation, position and rotation of the impacted tooth affected the duration of orthodontic treatment. Another study reported that the location of the canine subjected to forced eruption was a major factor in the total treatment duration and that the treatment duration increased as the distance from the occlusal surface increased [25]. For all the teeth investigated in the present study, the odds of an increase in the orthodontic treatment duration for more than 2 years were 1.967 times when the apex was closed, 3.903 times when the tooth was positioned buccally/palatally, and 3.628 times when the tooth was rotated horizontally. The age at which the apex closed based on the premolars is approximately 9 to 15 years, and in this study, the apex was found to be closed on radiographs from the age of 12. Age was not significant in the duration in this study, but considering the age at which apex formation is closed, it was found that it could be an important factor in the duration of orthodontic treatment. Several studies have reported that the treatment difficulty of impacted teeth is correlated with bucco-palatal position and horizontal position [26, 27]. The results of this study also showed that the treatment difficulty increases for teeth located in the bucco-palatal position rather than for teeth positioned in the line in the arch, and for teeth rotated horizontally rather than vertically, so it seems inevitable that the treatment duration will increase. Additionally, Grisar et al. showed that the average duration of orthodontic treatment in the re-operation group was 25 months, whereas in this study, it was 20.36 months [28]. this study showed that the duration of orthodontic treatment was shortened in re-operated teeth. If the teeth are not fully exposed during forced eruption, they may become impacted again or fail to erupt properly; therefore, re-operation may be required to correct this problem. The average duration from the first button attachment to re-operation was 6.20 months, indicating that the appropriate decision of re-operation reduced the treatment duration.

In this study, 25 (11.1%) of the 225 teeth that underwent forced eruption required re-operation, and most of them underwent reoperation due to button, ligature, or wire detachment. Similarly, Grisar et al. found that 19 (12%) of 153 canines that had undergone forced eruption during orthodontic treatment were re-operated because of a lack of movement, loose brackets, or wound infection [28]. These results highlight the importance of careful management and continuous monitoring during the forced eruption process.

In Cases 1–3, tooth extraction was performed after forced eruption failed due to ankylosis. Ankylosis is histologically defined as fusion of cementum/dentin to bone in at least one area resulting in loss of periodontal ligament space in that area [29, 30]. This diagnosis was established by analyzing dental radiographs and clinical information such as loss of tooth mobility. However, despite the well-known advantages of Cone Beam Computed Tomography (CBCT) for several diagnostic tasks in dentistry, he diagnosis of ankylosis in impacted teeth is still hindered by a limited approach to individual pulp and affected structures in the clinical practice [31]. These limitations make it difficult to diagnose ankylosis of impacted teeth based on only CBCT imaging due to limited access to the impacted tooth and its surrounding structures.

In Cases 2 and 3, the patients were in the growth stage, during which it is often necessary to extract the ankylosed tooth to prevent malocclusion aggravation, which can cause a lateral open bite to develop and inhibit vertical growth in the alveolar process. Another treatment option is to align the ankylosed teeth with orthodontic force; however, surgical intervention is required to do so because of the risk of root fractures, re-ankylosis, or damage to adjacent structures before the teeth are aligned in their normal positions in the dental arch [32, 33]. Therefore, a definite diagnosis of ankylosis is essential before treatment.

In Cases 5 and 6, forced eruption was performed for 18–24 months; however, the treatment failed. Although there was no evidence of ankylosis, tooth extraction was performed as the teeth had not moved for a long time. This is known as primary failure of eruption, in which non-ankylosed teeth do not erupt because of a malfunction in the eruption mechanism. The exact cause is unknown; however, genetic disorders with variable penetrance and expression are the most likely explanation [34].

A new measurement scale for impacted canines based on three different cone-beam computed tomography (CBCT) views was introduced to assess the difficulty of impaction and the potential efficacy of the treatment. Although CBCT is an effective tool for diagnosing and planning treatments for impactions, its clinical usefulness and reliability have not yet been evaluated [35]. In this study, a detailed CBCT measurement analysis was not included; however, a comprehensive study on the forced eruption of impacted teeth was performed by examining the entire impacted tooth.

This retrospective study investigated a relatively large sample of impacted teeth, including canines along with other teeth. Compared with previous studies focusing only on canines, this study provides a more comprehensive understanding of the outcomes of forced eruption treatment but has several limitations. First, due to the small number of failed samples, the study findings may not be generalized to other populations or dental practices. Second, because all teeth that undergone forced eruption were targeted, the characteristics of specific teeth could not be reflected. Therefore, further studies should address the limitations of this study by using a larger sample size and focusing on specific teeth to evaluate clinical or radiological outcomes to determine other factors that may affect forced eruption treatment outcomes.

## Conclusion

The success of forced eruption was only associated with age, but the treatment duration was statistically significant with open apex, bucco-palatal (lingual) position, rotation, and re-operation. The re-operation was success rate of 96% with 24 of 25 teeth being successful, and the treatment period was 20.36 ± 11.05 month. To increase the success rate of forced eruption, age should be considered as a priority, and in order to predict the treatment period, the apex formation status, position in the arch, and rotation should be considered in addition to age. When determining re-operation, considering factors such as ankylosis, root curvature, and apex formation can help in the success of orthodontic treatment.

### Electronic supplementary material

Below is the link to the electronic supplementary material.

Supplementary Material 1

## Data Availability

Data that support the findings of this study are available from the corresponding authors upon request and following IRB rules and privacy regulations.
