# Supplementary material for: Forced eruption in impacted teeth: analysis of failed cases and outcome of re-operation
Source: BMC Oral Health. 2024 Feb 20;24:254. doi: 10.1186/s12903-024-03963-x (PMC10877739; doi:10.1186/s12903-024-03963-x)
Supplement: Supplementary file 1 — Supplementary Material 1 [file 12903_2024_3963_MOESM1_ESM.docx]

**Forced Eruption in Impacted Teeth:**

**Analysis of Failed Cases and Outcome of Re-operation**

Jaeyeon Kim^*^, Seoyeon Jung^†^, Kee-Joon Lee^‡^, Hyung-Seog Yu^‡^, Wonse Park*

^*^Department of Advanced General Dentistry, College of Dentistry, Yonsei University, Seodaemun-gu, Seoul, South Korea

^†^Department of Dental Education, College of Dentistry, Yonsei University, Seodaemun-gu, Seoul, South Korea

^‡^Department of Orthodontics, Institute of Craniofacial Deformity, College of Dentistry, Yonsei University, Seodaemun‑gu, Seoul, South Korea

Corresponding author: Wonse Park

Address: Department of Advanced General Dentistry, College of Dentistry, Yonsei University, 50-1 Yonsei-ro, Seodaemun-gu, Seoul 03722, South Korea

Tel: +82 2-2228-8985

Email: wonse@yuhs.ac

*Case descriptions of forced eruption failure*

*Case descriptions of forced eruption failure*

**Case descriptions of forced eruption re-operation and failure**

**Re-operation case 1**: A 23-year-old female presented with an impacted lower left second premolar tooth, with the crown impacted proximal to the root of the first molar. It was found that the root was slightly curved in the direction of orthodontic movement. To perform forced eruption, a wire was exposed and attached to the mesial buccal gingiva. Six months after forced eruption, there was no remarkable movement due to bone remodeling. Subsequently, an ostectomy was performed on the upper part of the crown to induce tooth movement for rapid tooth movement. Six months after the re-operation, orthodontic extrusion was confirmed, and forced eruption and orthodontic treatment were successfully completed (Figure 1).

**Failure case 1**: A 24-year-old woman presented with a complete horizontal impaction of the maxillary right canine with the crown on the mesial side and root on the distal side. A forced eruption was performed by attaching a button close to the palatal surface cusp tip and positioned the ligature in the direction of the palatal pin placed between the maxillary right first and second molars. After three months, ankylosis on the buccal and lingual sides was confirmed, and no movement was observed. One month after the ankylosis was confirmed, tooth extraction was performed, and orthodontic treatment was completed after 29 months (Figure 2).

**Failure case 2**: A 12-year-old girl presented with an impacted maxillary second premolar whose crown was at the center of the lingual surface of the first premolar root, and the root was located distal to the first premolar root. A button was attached to the lingual cervical of the second premolar. Forced eruption was induced by exposing the wire to the palatal mucosa. After nine months, tooth extraction was performed because of difficulty in orthodontic repositioning. Orthodontic treatment was completed after 45 months (Figure 3).

**Failure case 3**: A 9-year-old girl was treated for forced eruption of the mandibular right first molar. Considering the amount of attached gingiva, only one cusp was opened and a button was attached. After 12 months, ankylosis was confirmed and tooth extraction was performed. After approximately two years, orthodontic treatment was completed by uprighting the second molar (Figure 4).

**Failure case 4**: A 29-year-old woman had a left mandibular canine that did not erupt even after the adjacent tooth was extracted. Forced eruption was performed; however, one month later, reopening and traction were performed due to button detachment. No movement was observed after monthly follow-ups for approximately two years. After tooth extraction, an implant was placed and orthodontic treatment was completed (Figure 5).

**Failure case 5**: A 45-year-old male presented with an impacted left maxillary canine. For distal traction of the impacted canine crown, the crown of the canine was opened to perform forced eruption and opening of the orthodontic space. After 18 months, space opening was achieved, but the eruption of the impacted canine failed; therefore, tooth extraction was performed. After a healing duration of 2–3 months, implants were placed, and orthodontic treatment was completed after six months (Figure 6).

**Failure case 6**: A 13-year-old girl presented with impacted left and right maxillary canines. The crown of the maxillary right canine was located at the center of the buccal root of the maxillary right first molar, and the root was located at the root apex of the maxillary right first premolar. The maxillary right canine was impacted far from the normal eruption path, making forced eruption difficult; therefore, extraction was performed without forced eruption (Figure 7).

**
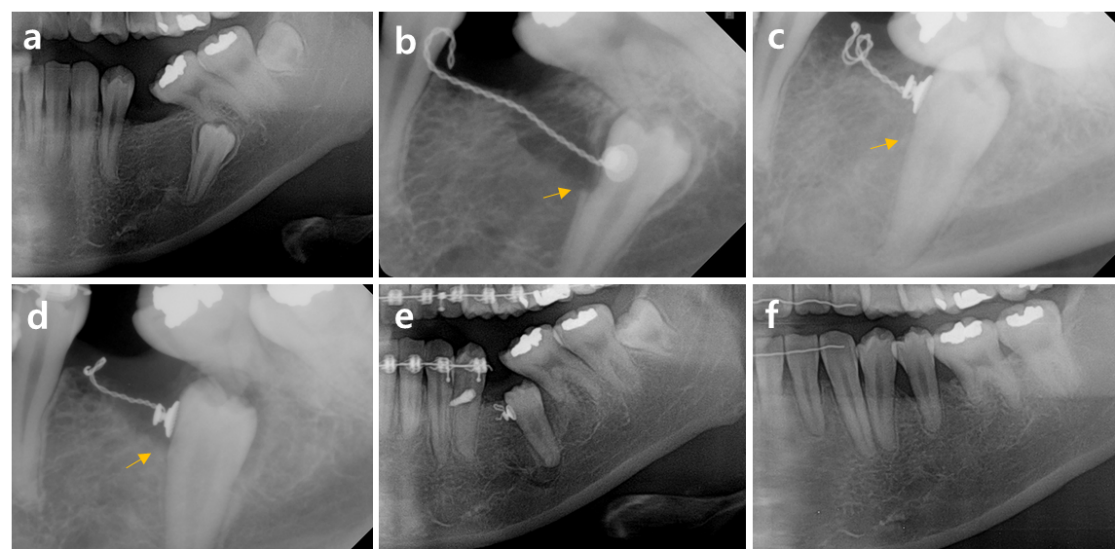
**

**Figure 1** Re-operation case 1. (a) mandibular left second premolar is close to the root of the first molar; (b) forced eruption is performed by exposing the wire to the mesio-buccal gingiva; (c) no significant movement after 6 months of forced eruption; (d) ostectomy was performed due to bone remodeling; (e) observation of orthodontic extrusion after re-operation; (f) completion of orthodontic treatment.

**
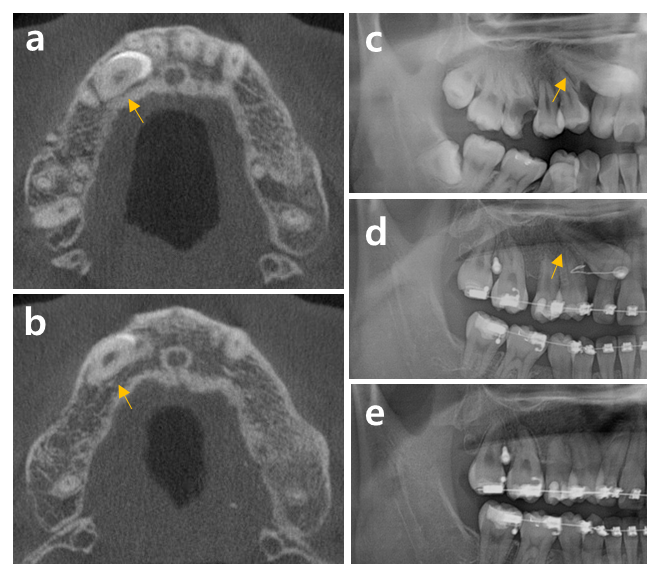
**

**Figure 2** Failure case 1. (a, b, c) horizontally impacted maxillary right canine; (d) no movement was observed and ankylosis was confirmed; (e) orthodontic treatment state after tooth extraction.

**
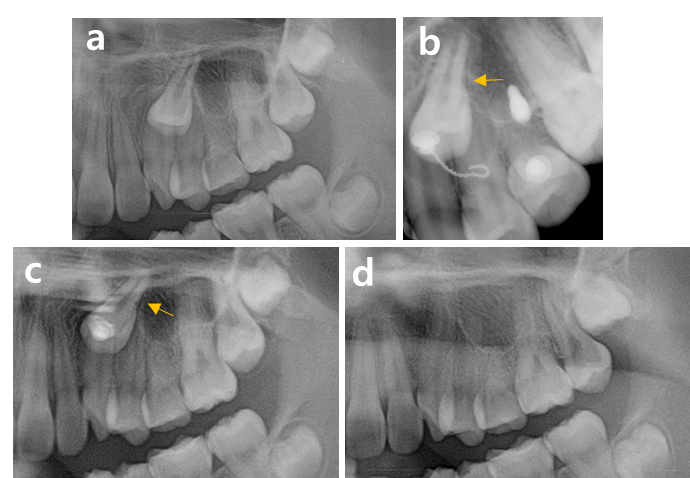
**

**Figure 3** Failure case 2. (a) vertical impacted maxillary left second premolar; (b, c) forced eruption was performed but ankylosis was confirmed after 9 months; (d) completion of orthodontic treatment after surgical extraction.

**
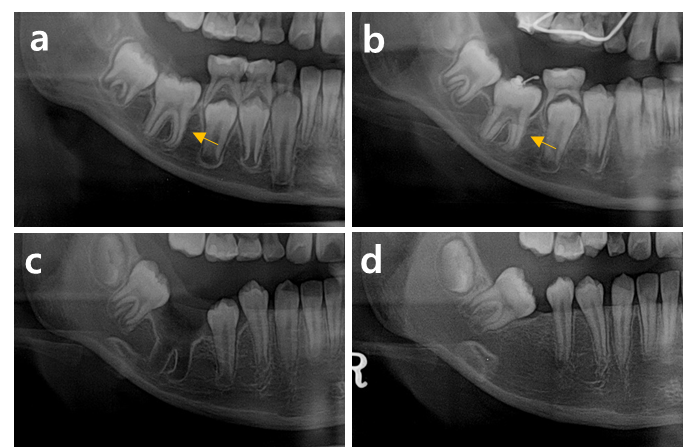
**

**Figure 4** Failure case 3. (a) fully impacted mandibular right first molar; (b) no tooth movement and ankylosis was confirmed; (c) surgical extraction; (d) uprighting the mandibular right second molar.

**
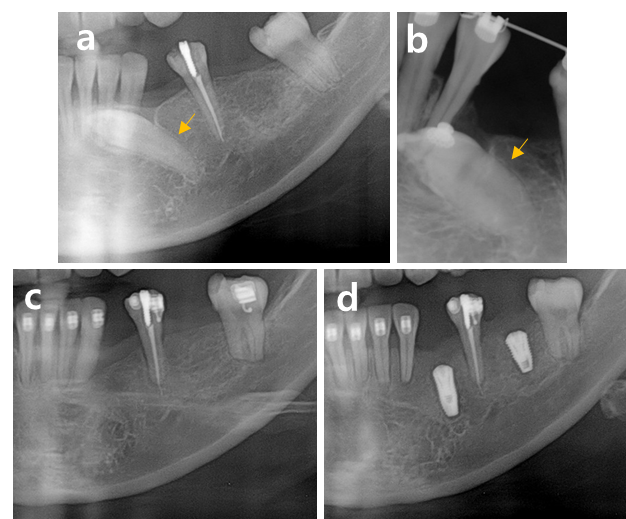
**

**Figure 5** Failure case 4. (a, b) mandibular left canine that did not erupt even after extraction of the mandibular left first premolar; (c) no movement observed 2 years after re-operation; (d) proceed with orthodontic treatment and implant placement.

**
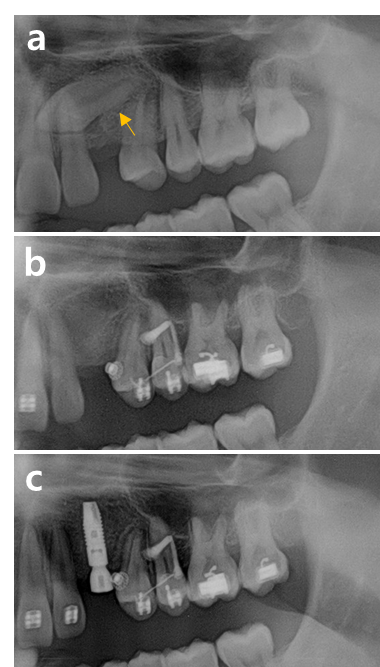
**

**Figure 6** Failure case 5. (a, b) after 18 months, space formed but the impacted canine did not erupt and so was extracted; (c) after 3 months, implants were placed and orthodontic treatment was performed.

**
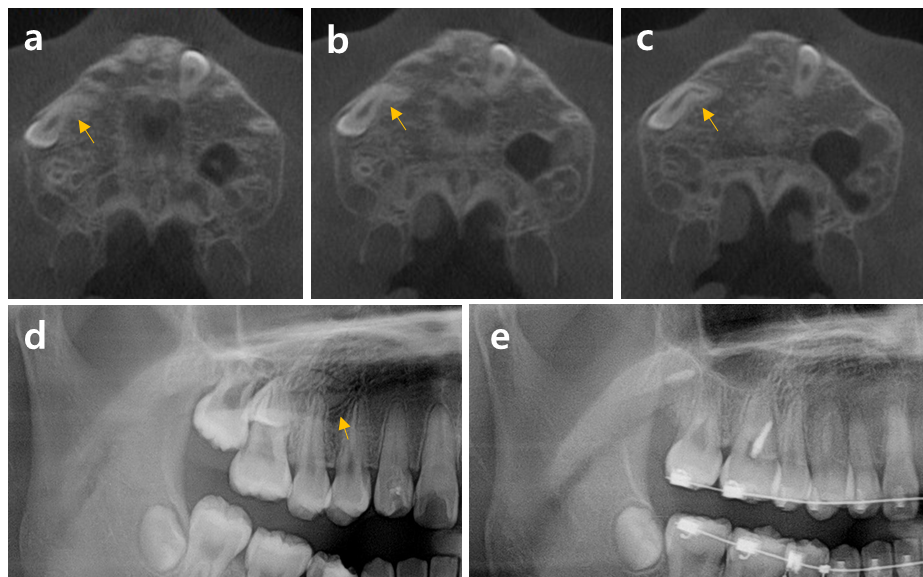
**

**Figure 7** Failure case 6. (a, b, c) The maxillary right canine was shown to be too far from the dental arch by cone-beam computed tomography radiography; (d) The ectopically fully impacted maxillary right; (e) Orthodontic treatment state after tooth extraction.
